# Quantum Nonseparability Without Nonlocality: A *ψ*-Ontic Holistic Account of Entangled Measurement

**DOI:** 10.3390/e28091009

**Published:** 2026-09-09

**Authors:** Everett X. Wang

**Affiliations:** Beijing Institute of Nanoenergy and Nanosystems, Chinese Academy of Sciences, Beijing 101400, China; wangxiaolin@binn.cas.cn

**Keywords:** quantum entanglement, nonlocality, nonseparability, *ψ*-ontic, wavefunction realism, Bell’s theorem, decoherence, continuous spontaneous localization, holism, measurement problem

## Abstract

The standard interpretation of quantum measurement on entangled systems holds that measuring one particle nonlocally collapses the wavefunction of its spacelike-separated partner. We argue that this conclusion rests on a false presupposition: that subsystems of entangled systems possess independent ontic states. If the global wavefunction is the sole ontic object (ψ-ontic holism), then for entangled systems, there is no “state of B” to be affected by measurement at A. Measurement is a local dynamical process—concretely modeled by continuous spontaneous localization—that destroys one local wavefunction component at the measurement site; the global state factorizes as a consequence, and subsystem ontology emerges for the first time. The transition of the distant particle’s reduced density matrix from mixed to pure reflects this emergence of separability, not a physical change at the distant location. The framework satisfies no-signaling and embraces the contextuality required by the Kochen–Specker and GHZ theorems. We are explicit about its relation to Bell’s theorem: Bell local causality (factorizability) fails, as it must in any empirically adequate theory, but the failure is confined to outcome independence and is identified with the nonseparability of the global ontic state, while parameter independence—and with it the locality of the dynamics—holds exactly. Decoherence provides the mechanism by which the global wavefunction factorizes and classical separability emerges. The apparent nonlocality of quantum mechanics is thus reinterpreted as nonseparability: the fundamental ontology is holistic, but the dynamics are local.

## 1. Introduction

Since the celebrated Einstein–Podolsky–Rosen (EPR) argument [1] and Bell’s subsequent theorem [2], the consensus in quantum foundations has been that quantum mechanics is irreducibly nonlocal: measuring one half of an entangled pair instantaneously affects the physical state of its distant partner. This view is reinforced by the textbook projection postulate, which states that upon measurement of observable $\hat{A}$ on subsystem A of an entangled state ${|\Psi \rangle }_{AB}$, the global state undergoes instantaneous projection irrespective of the spatial separation between A and B.

The discomfort with this conclusion is well known. Nonlocal collapse sits uneasily with special relativity, which prohibits superluminal causal influence. While the no-signaling theorem [3,4] ensures that no exploitable information is transmitted, the ontological status of the distant state change remains deeply problematic. Does the wavefunction of particle B really change when particle A is measured? If so, what mediates this change across spacelike separation? If not, what licenses the standard Copenhagen account?

In a companion paper [5], we proposed Postulate M, a local measurement axiom that replaces the nonlocal projection postulate with a local condition tied to decoherence: a single outcome is realized at the measurement site when local decoherence is complete, without reference to distant subsystems. That paper was deliberately agnostic about ontological commitments, presenting Postulate M as a structural axiom compatible with multiple interpretations. In a related work [6], we modeled measurement as a local system–environment interaction within the decoherence framework; the corrected analysis of that model, presented in [5], shows that the maximal Bell–CHSH value evolves as $S_{\max}\left(t\right)=2\sqrt{1+{\left|z\left(t\right)\right|}^{2}}$, decaying smoothly from the Tsirelson bound $2\sqrt{2}$ to the classical bound 2 as decoherence completes, while the outcome probabilities in the measurement basis are exactly preserved at all times, confirming that quantum correlations are structural features of the initial entangled state, progressively consumed by local environmental entanglement rather than maintained or produced by any nonlocal influence. This decay has a direct ontological meaning in the framework developed below: as long as the global state remains non-factorizable, the subsystems possess no independent ontic states, and the excess of $S_{\max}$ over the classical bound is the observable signature of that holism; only when decoherence completes does the global wavefunction factorize, whereupon independent subsystem states emerge for the first time and the maximal correlation settles at the classical bound.

Throughout this paper, “ontic” is used in the sense of the ontological-models framework [7]: a mathematical object is ontic if it represents (or is determined by) the physical state of affairs itself as opposed to “epistemic” objects that represent knowledge, information, or predictive bookkeeping. The question we press is *which* object in the quantum formalism deserves ontic status for entangled composites. Our answer—only the global wavefunction—places this work within a broader landscape of responses to quantum nonlocality: Howard’s reconstruction of Einstein’s separability principle [8], Teller’s relational holism [9], d’Espagnat’s analysis of improper mixtures [10], Maudlin’s defense of genuine nonlocality [11], Norsen’s analysis of local causality [12], relational quantum mechanics [13], Everettian approaches [14], and operational reconstructions that deflate nonlocality talk [15]. Section 9 engages these positions in detail; we flag them here so the reader can situate the proposal from the outset.

The present paper provides the ontological foundation for Postulate M. We argue that the appearance of nonlocal measurement arises from a category error: the false presupposition that subsystems of entangled systems possess independent ontic states. Our central claims are:

(i) The global wavefunction is the sole ontic object for entangled systems (ψ-ontic holism). Subsystem wavefunctions and reduced density matrices are calculational tools, not descriptions of independent reality.
(ii) Measurement is a local dynamical process that modifies only local wavefunction components. Continuous spontaneous localization (CSL) provides a concrete model: a nonlinear localization term and noise fields at the measurement site destroy one local component, causing the global state to factorize.
(iii) Factorization—not collapse at B—is what creates subsystem ontology. Only after the global state becomes separable can we meaningfully speak of “B’s state.” The reduced density matrix transition from mixed to pure reflects this emergence, not a physical change at B’s location.
(iv) Bell’s theorem, in its general stochastic form, rests on the factorizability (local causality) condition, which decomposes into parameter independence and outcome independence [12,16,17,18,19]. Our framework preserves parameter independence exactly—the dynamics contain no action at a distance—and locates the entire failure of factorizability in outcome independence, which we identify with the nonseparability of the global wavefunction rather than with any dynamical influence. Independently, the Kochen–Specker and GHZ theorems rule out noncontextual pre-assigned values, blocking the EPR route from perfect correlations to determinism; but we emphasize that rejecting such values is not, by itself, an escape from Bell’s theorem.
(v) Decoherence is ubiquitous and provides the universal mechanism for factorization. Classical separability—the appearance of independent objects with definite properties—is emergent rather than fundamental.

The paper is organized as follows. Section 2 reviews the standard nonlocality argument and identifies its implicit presupposition. Section 3 presents our ψ-ontic holistic ontology. Section 4 analyzes measurement as a local process using the CSL framework. Section 5 addresses the reduced density matrix and argues that it is not ontic for entangled systems. Section 6 analyzes Bell’s theorem in its stochastic (factorizability) form, locates its failure in outcome independence, and presents the Kochen–Specker and GHZ results. Section 7 provides a formal mathematical framework with definitions and theorems. Section 8 discusses decoherence and the emergence of separability. Section 9 situates our view relative to existing interpretations and philosophical work on quantum holism. Section 10 concludes.

## 2. The Standard Nonlocality Argument and Its Hidden Presupposition

### 2.1. The Standard Account

Consider the canonical EPR–Bohm scenario. Two spin-$\frac{1}{2}$ particles are prepared in the singlet state:

(1) $$|\Psi \rangle =\frac{1}{\sqrt{2}}\left({|↑\rangle }_{A}⊗{|↓\rangle }_{B}-{|↓\rangle }_{A}⊗{|↑\rangle }_{B}\right),$$

where ${|↑\rangle }_{A}$ and ${|↓\rangle }_{A}$ denote the spin-up and spin-down states for particle A, and similarly for B. The singlet state has total spin angular momentum zero; the anticorrelation between the two particles’ spins is a direct consequence of angular momentum conservation during the pair-creation process. The particles separate and travel to spacelike-separated measurement stations.

The standard account proceeds as follows. Before measurement, particle B’s reduced density matrix is:

(2) $$\rho_{B}={\mathrm{Tr}}_{A}\left(|\Psi \rangle \langle \Psi |\right)=\frac{1}{2}{|↑\rangle }_{B}{\langle ↑|}_{B}+\frac{1}{2}{|↓\rangle }_{B}{\langle ↓|}_{B}\;.$$

This is a maximally mixed state. After Alice measures particle A and obtains spin-up, the global state collapses to:

(3) $$|\Psi \rangle \;\to {\;|↑\rangle }_{A}⊗{|↓\rangle }_{B}\;,$$

and B’s reduced density matrix becomes:

(4) $$\rho_{B}={|↓\rangle }_{B}{\langle ↓|}_{B}\;.$$

This is a pure state. The transition $\rho_{B}:\mathrm{mixed}\to \mathrm{pure}$ is taken as evidence that measurement at A physically affected B’s state—a nonlocal influence across spacelike separation.

### 2.2. The Hidden Presupposition

The standard argument implicitly assumes that the reduced density matrix $\rho_{B}$ describes the *ontic state* of particle B—that is, that $\rho_{B}$ represents a real, independently existing physical state of the subsystem. This assumption is so deeply embedded in the formalism that it is rarely examined. But it is precisely this assumption that generates the appearance of nonlocality.

If $\rho_{B}$ is ontic, then its change from mixed to pure constitutes a real physical change at B’s location, caused by a measurement at A—nonlocal action. But if $\rho_{B}$ is *not* ontic for entangled systems—if particle B simply does not have an independent physical state while entangled with A—then there is nothing at B to change, and the nonlocality argument collapses.

This is the category error at the heart of the standard account: it asks “what happened to B’s state?” while presupposing that B has an independent state. For entangled systems, this presupposition is false.

### 2.3. Three Senses of Locality

Before proceeding, we fix terminology, since “locality” names at least three inequivalent conditions whose conflation has generated much confusion:

(L1) **No-signaling:** Bob’s unconditioned outcome statistics are independent of Alice’s choice of measurement (and of whether she measures at all).
(L2) **Dynamical locality:** every dynamical element of the theory—Hamiltonians, decoherence couplings, collapse operators, noise fields—has support only in a bounded spatial region; equivalently, at the level of an ontological description, *parameter independence* holds: the response of each wing is statistically independent of the distant *setting*.
(L3) **Bell local causality (factorizability):** in an ontological model with complete state λ, settings x,y and outcomes a,b, P(a,b|x,y,λ)=P(a|x,λ)P(b|y,λ), together with measurement-setting independence of λ.

These are strictly ordered in strength: (L3) implies (L2) implies (L1), and the converses fail. Our claims in this paper are precisely these: quantum mechanics, read through ψ-ontic holism, satisfies (L1) and (L2); it violates (L3), and *must* violate it, by Bell’s theorem. With λ=|Ψ〉 taken as the complete ontic state, the factorization in (L3) fails through its outcome-independence factor, while its parameter-independence factor holds exactly (Section 6.7). The thesis of the title—“nonseparability without nonlocality”—is therefore the claim that the (L3)-violation is wholly accounted for by the nonseparability of λ itself, with no failure of (L2): no dynamical influence, no action at a distance, no preferred frame. Whenever we deny “nonlocality” below, it is dynamical nonlocality—the failure of (L2)—that is denied; we do not dispute, and no consistent reading of Bell permits disputing, the failure of (L3).

## 3. ψ-Ontic Holism: The Global Wavefunction as Sole Ontic Object

### 3.1. The Ontological Claim

We adopt a ψ-ontic ontology [7,20]: the wavefunction represents physical reality, not merely information or belief. The PBR theorem [20] shows that, within the ontological-models framework and under a preparation-independence assumption, the quantum state of a system cannot be merely epistemic: distinct pure states correspond to disjoint ontic-state distributions. We take PBR as strong *motivation* for wavefunction realism, while acknowledging plainly what it does not establish: it does not prove global wavefunction monism, does not show that reduced density operators are non-ontic, and does not select configuration-space realism. Those further commitments are argued for on independent grounds in this paper, not derived from PBR. We accept this conclusion at the level of the *global* wavefunction: distinct global states ${|\Psi \rangle }_{AB}$ and ${|\Phi \rangle }_{AB}$ correspond to distinct physical realities. However, we deny that the PBR result extends to reduced density matrices of entangled subsystems, since these are derived quantities obtained by partial trace—an operation whose ontological legitimacy we question for non-factorizable states. The reduced state $\rho_{B}$ is not a quantum state in the PBR sense (it is not a preparation of system B), but rather a mathematical summary of correlations within the global state.

But we make a further commitment that distinguishes our view from generic wavefunction realism: for entangled systems, only the global wavefunction ${|\Psi \rangle }_{AB}$ is ontologically real. The subsystem wavefunctions, reduced density matrices, and local “states” are derived quantities that do not independently represent physical reality.

This is not a novel metaphysical position; it is forced by the mathematical structure of entanglement. For the singlet state:

(5) $$|\Psi \rangle =\frac{1}{\sqrt{2}}\left({|↑\rangle }_{A}⊗{|↓\rangle }_{B}-{|↓\rangle }_{A}⊗{|↑\rangle }_{B}\right),$$

there exist no states $|\psi_{A}\rangle$ and $|\psi_{B}\rangle$ such that $\left|\Psi \rangle =\right|\psi_{A}\rangle ⊗|\psi_{B}\rangle$. The state is non-factorizable. This is not a limitation of our knowledge; it is a structural property of the wavefunction. The system AB is an irreducible whole.

### 3.2. What “Holism” Means Operationally

Our holistic ontology has precise operational consequences:

(a) **No independent subsystem properties.** For an entangled system, the question “what is the spin of particle B?” has no answer prior to measurement. This is not because the answer is unknown (hidden variables), but because the question is ill-posed: B does not have an independent spin state. Properties are relational features of the global wavefunction, not intrinsic features of subsystems.
(b) **The reduced density matrix is a calculational tool.** $\rho_{B}={\mathrm{Tr}}_{A}(|\Psi \rangle \langle \Psi |)$ is computed by tracing out A’s degrees of freedom—an operation that presupposes that B can be described independently. For entangled systems, this presupposition is false. $\rho_{B}$ is operationally useful (it correctly predicts measurement statistics at B), but it does not describe B’s ontic state.
(c) **Separability requires factorizability.** Any composite system—whether entangled or not—is described by a state in the tensor product Hilbert space $H_{A}⊗H_{B}$. However, only when the global state can be written as a simple separable product $\left|\Psi \rangle =\right|\psi_{A}\rangle ⊗|\psi_{B}\rangle$ (i.e., when the state factorizes) does it become meaningful to attribute independent ontic states to the subsystems. For entangled states, the state lies in $H_{A}⊗H_{B}$ but cannot be so factorized; the subsystems do not possess independent states despite inhabiting a tensor product space.

### 3.3. Einstein’s Moon and ψ-Ontic Realism

Einstein famously asked: “Is the Moon there when nobody looks?” His question expressed a deep commitment to objective reality—the conviction that physical objects possess definite properties independently of observation. The Copenhagen interpretation answered negatively for quantum systems: properties come into being only upon measurement, and the wavefunction merely encodes an observer’s knowledge.

Our ψ-ontic holism offers a different answer that honors Einstein’s realist intuition while remaining faithful to quantum mechanics. In our framework, the Moon is indeed “there” when nobody looks—but what is “there” is the wavefunction, not a classical object with predetermined measurement outcomes. The Moon, as a macroscopic object thoroughly decohered by its environment, exists in a factorized state with definite pointer-state properties (position, shape, reflectivity). Its wavefunction is real and objective, independent of any observer. No one needs to look at the Moon for it to possess its physical state.

The subtlety arises for microscopic quantum systems. For a single spin in superposition, our framework says: the wavefunction α|↑〉+β|↓〉 is objectively real—it is “there”—but it does not correspond to a definite spin value in any direction. The spin observable does not have a pre-existing value waiting to be revealed; rather, measurement (via local decoherence) factorizes the system-apparatus-environment wavefunction and produces a definite outcome. Reality exists before measurement—it is the wavefunction—but definite measurement outcomes emerge through the local physical process of decoherence and factorization.

Thus, our framework restores Einstein’s realism at a deeper level: the wavefunction is the objective element of reality, present whether or not anyone observes it. What Einstein could not accept—and what our framework dissolves—is the apparent nonlocality of measurement. In our account, measurement is local, reality is the global wavefunction, and the Moon is always there.

### 3.4. Configuration Space Realism

Our ontology naturally implies configuration-space realism: the wavefunction $\Psi (x_{A},x_{B})$ lives in the 6-dimensional configuration space of the two-particle system, not in 3-dimensional physical space. The two particles, although spatially separated in 3D, are described by a single field in configuration space. “Spacelike separation” in 3D does not imply separation in configuration space—the wavefunction is a single connected entity.

This perspective dissolves the mystery of entanglement. Two particles are entangled precisely when the configuration-space wavefunction does not factorize. The correlations between measurements at A and B are not transmitted across 3D space; they are encoded in the structure of the configuration-space wavefunction from the moment of preparation.

We acknowledge that configuration-space realism carries significant philosophical costs. Critics such as Maudlin [11] have argued that configuration space cannot be fundamental because its very definition presupposes the 3D spatial structure it is supposed to explain: configuration space is the space of possible *spatial* configurations of particles, so 3D space appears to be conceptually prior. We take this objection seriously but note that it applies equally to any wavefunction-realist interpretation. Our position is that 3D spatial structure may emerge from the configuration-space wavefunction through decoherence and factorization—the same mechanism that generates classical separability—rather than being presupposed. A full treatment of this emergence lies beyond the scope of the present paper.

## 4. Measurement as a Local Process

### 4.1. The Local Dynamics of Collapse

In our framework, measurement at A is a local dynamical process: it modifies only the wavefunction components that have support in A’s spatial region, without affecting components at B’s location.

To make this concrete, we employ the Continuous Spontaneous Localization (CSL) model [21,22] as an illustrative mechanism. In CSL, the Schrödinger equation is supplemented with nonlinear stochastic terms:

(6) $$d|\psi_{t}\rangle =\left[-\frac{i}{\hbar }\hat{H}\;dt-\frac{\lambda }{2}{\left(\hat{A}\left(x\right)-{\langle \hat{A}\left(x\right)\rangle }_{t}\right)}^{2}dt+\sqrt{\lambda }\left(\hat{A}\left(x\right)-{\langle \hat{A}\left(x\right)\rangle }_{t}\right)dW_{t}\left(x\right)\right]|\psi_{t}\rangle ,$$

where $\hat{A}\left(x\right)$ is the collapse operator, λ is the collapse rate, and $dW_{t}\left(x\right)$ is a Wiener noise field.

Equation (6) is a deliberately schematic, normalized (nonlinear) form, and three clarifications are required to connect it to standard CSL [21,22]. First, in standard mass-proportional CSL, the collapse operators are smeared mass-density operators $\hat{M}\left(x\right)$, smeared over the correlation length $r_{C}≃10^{-7}$ m, with a spatial integral over x and a white-noise field w(x,t); CSL does not directly collapse an arbitrary spin observable. In a Stern–Gerlach measurement the spin must first be correlated, by ordinary unitary dynamics, with spatially distinct macroscopic pointer configurations; only then does the mass-density mechanism act on the pointer. Our schematic $\hat{A}\left(x\right)$ should be read as the effective collapse operator induced on the measured system through this pointer correlation. Second, the theory exists at three levels that must not be conflated: the *linear* unnormalized stochastic equation, whose solutions define raw branch weights; the *nonlinear normalized* Equation (6), describing individual trajectories; and the ensemble average, which is a linear completely positive trace-preserving (Lindblad) evolution. Third, the amplification of the effective rate for a macroscopic pointer is not universally $N^{2}\lambda$: the scaling depends on the object’s mass distribution relative to $r_{C}$ (it is quadratic in the number of constituents displaced coherently by more than $r_{C}$, with well-known geometric corrections). We use $\lambda_{\mathrm{eff}}∼N^{2}\lambda$ only as the standard order-of-magnitude statement for a rigid pointer displaced beyond $r_{C}$.

The crucial feature for our purposes is that the nonlinear collapse term and the noise field couple to the system through operators supported in A’s spatial region. When particle A interacts with a measurement apparatus (a macroscopic object with $N∼10^{23}$ particles), the effective collapse rate is amplified, driving rapid localization of A’s pointer components. The CSL noise field is universal—it is present at B’s location as well—but with no macroscopic pointer at B there is no amplification, and the unamplified single-particle rate ($\lambda ≃10^{-17}\;s^{-1}$ in the GRW parameter choice) is utterly negligible on all relevant timescales; B’s wavefunction components are, to this accuracy, unaffected by the dynamics.

### 4.2. Tracing Through an EPR Measurement

**Figure 1:**
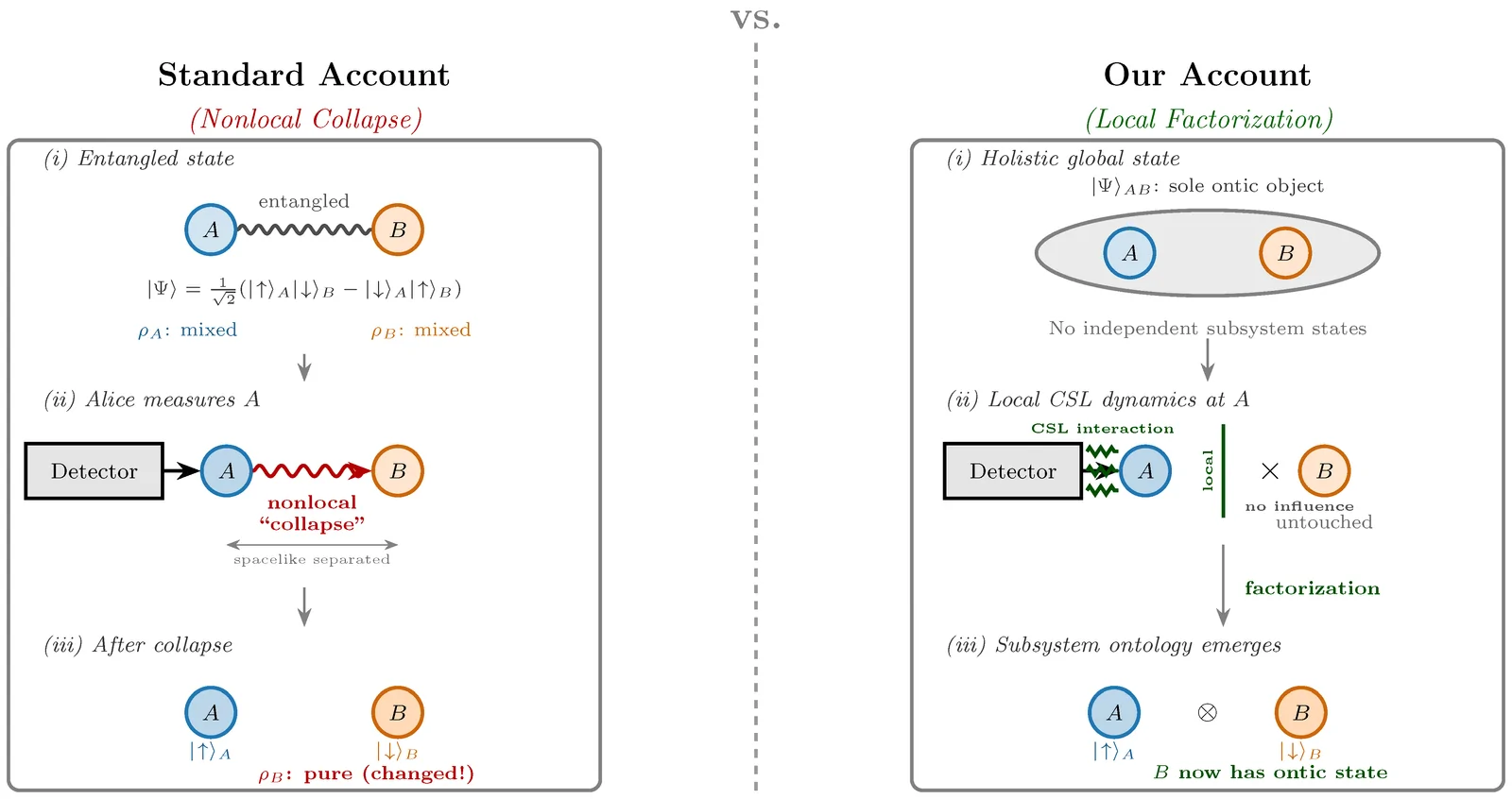
Comparison of the standard account (**left**) and our account (**right**) of EPR measurement. In the standard account, measurement at A nonlocally collapses B’s state across spacelike separation. In our account, local CSL dynamics at A destroy one wavefunction component; the global state factorizes, and subsystem ontology for B emerges without any nonlocal influence.

Consider the singlet state $|\Psi \rangle =\frac{1}{\sqrt{2}}{(|↑\rangle }_{A}{|↓\rangle }_{B}-{|↓\rangle }_{A}{|↑\rangle }_{B})$. When Alice performs a spin-*z* measurement:

**Step 1: Local interaction.** Particle A enters a Stern–Gerlach apparatus. The apparatus (macroscopic) becomes entangled with A:
  (7) $$|\Psi \rangle \;\to \;\frac{1}{\sqrt{2}}\left({|↑\rangle }_{A}|D_{↑}{\rangle |↓\rangle }_{B}-{|↓\rangle }_{A}|D_{↓}{\rangle |↑\rangle }_{B}\right),$$
  where $|D_{↑}\rangle$ and $|D_{↓}\rangle$ are macroscopically distinguishable detector states. This step is unitary and local—the Hamiltonian couples only A to its local apparatus.
**Step 2: Local decoherence and CSL collapse.** The macroscopic detector undergoes rapid decoherence via interaction with its local environment, followed by CSL-driven localization. The nonlinear localization term and the stochastic noise at A’s location together drive one component (say ${|↓\rangle }_{A}|D_{↓}\rangle$) to zero amplitude: the deterministic term suppresses the component whose expectation value is disfavored, while the stochastic term provides the randomness that selects which component survives. This is a local process: only the wavefunction components with support in A’s spatial region are affected.
**Step 3: Factorization.** With one term eliminated, the global state becomes:
  (8) $$|\Psi \rangle \;\to {\;|↑\rangle }_{A}|D_{↑}{\rangle ⊗|↓\rangle }_{B}\;.$$
  The state has factorized. For the first time, it is meaningful to speak of “B’s state” as ${|↓\rangle }_{B}$. We stress the formal status of this step: it describes a single *selective* trajectory—the record of one realized outcome under the nonlinear normalized dynamics. It is not the nonselective (ensemble) transformation, which averages over the noise and is a linear completely positive map that leaves B’s marginal exactly unchanged (Theorem 4 below). The selective and nonselective descriptions answer different questions and must never be interchanged; Section 7 makes the distinction explicit. Figure 1 contrasts this account with the standard nonlocal-collapse account.

### 4.3. What Changed and What Didn’t

In this account:

- **What changed locally at A:** The wavefunction components ${|↑\rangle }_{A}|D_{↑}\rangle$ and ${|↓\rangle }_{A}|D_{↓}\rangle$ were driven to definite amplitudes (1 and 0) by the local nonlinear and stochastic dynamics.
- **What changed globally:** The global wavefunction went from non-factorizable (entangled) to factorizable (separable). This is a change in the global structure, caused by local dynamics at A.
- **What did not change at B:** The spin states ${|↑\rangle }_{B}$ and ${|↓\rangle }_{B}$—the physical degrees of freedom at B’s location—were completely unaffected. No noise acted on them. No signal propagated to B. No local observable at B changed.

### 4.4. CSL as Illustration, Not Commitment

We emphasize that CSL serves as a concrete illustration of how local dynamics can produce collapse, but our argument does not depend on CSL being the correct collapse mechanism. Any model in which measurement-induced collapse involves local dynamics at the measurement site—including gravitationally induced collapse models [23,24], decoherence-based approaches [25], or the local measurement axiom of Postulate M [5]—supports the same conclusion. The essential point is structural: local modification of wavefunction components at A’s location suffices to factorize the global state.

## 5. The Reduced Density Matrix Is Not Ontic for Entangled Systems

### 5.1. The Strongest Objection

The most powerful objection to our account is straightforward: “The reduced density matrix $\rho_{B}$ changed from mixed to pure. A physical quantity changed. Therefore something physical happened at B.”

We address this objection by distinguishing between operational utility and ontological status.

### 5.2. Operational vs. Ontological

The reduced density matrix $\rho_{B}={\mathrm{Tr}}_{A}(|\Psi \rangle \langle \Psi |)$ is the unique operator reproducing the expectation values of all observables in B’s local algebra, $\mathrm{Tr}\left(\rho_{AB}\;I_{A}⊗{\hat{O}}_{B}\right)=\mathrm{Tr}\left(\rho_{B}{\hat{O}}_{B}\right)$. We emphasize that this construction is mathematically valid precisely when A and B are entangled and presupposes no ontological independence of B whatsoever. The question we raise is therefore not about the legitimacy of the partial trace but about the *interpretive status* of its output: whether the operator so defined is a bearer of independent physical reality or a predictively complete piece of bookkeeping. Our answer—that for non-factorizable global states it is the latter—is a substantive ontological postulate, motivated below, and not a consequence of the mathematics alone; density-operator realism, algebraic local-state approaches, Everettian relative states, and relational accounts each make a different admissible choice at exactly this point.

$\rho_{B}$ is operationally useful: it correctly predicts the statistics of any local measurement performed on B alone. But operational utility does not entail ontological status. A probability distribution over hidden variables is operationally useful in Bayesian reasoning without the distribution itself being a physical entity.

For entangled systems, $\rho_{B}$ is an *improper mixture*—a term introduced by d’Espagnat [10] to distinguish the reduced state obtained by partial trace from a proper mixture reflecting genuine classical ignorance. An improper mixture cannot be interpreted as “B is in state ${|↑\rangle }_{B}$ with probability $\frac{1}{2}$ or ${|↓\rangle }_{B}$ with probability $\frac{1}{2}$.” The system is not in either state; it is part of an irreducible whole.

### 5.3. The Transition Reinterpreted

When $\rho_{B}$ transitions from mixed to pure upon measurement of A, the standard interpretation says: “B’s physical state changed.” Our interpretation says: “Before measurement, B had no independent physical state. After measurement—which caused factorization via local dynamics at A—B acquires an independent physical state for the first time.”

The transition $\rho_{B}:\mathrm{mixed}\to \mathrm{pure}$ does not represent a physical change at B. It represents the *emergence* of subsystem ontology through factorization. Before factorization, $\rho_{B}$ was a calculational device. After factorization, $\rho_{B}$ coincides with a genuine physical state.

**Figure 2:**
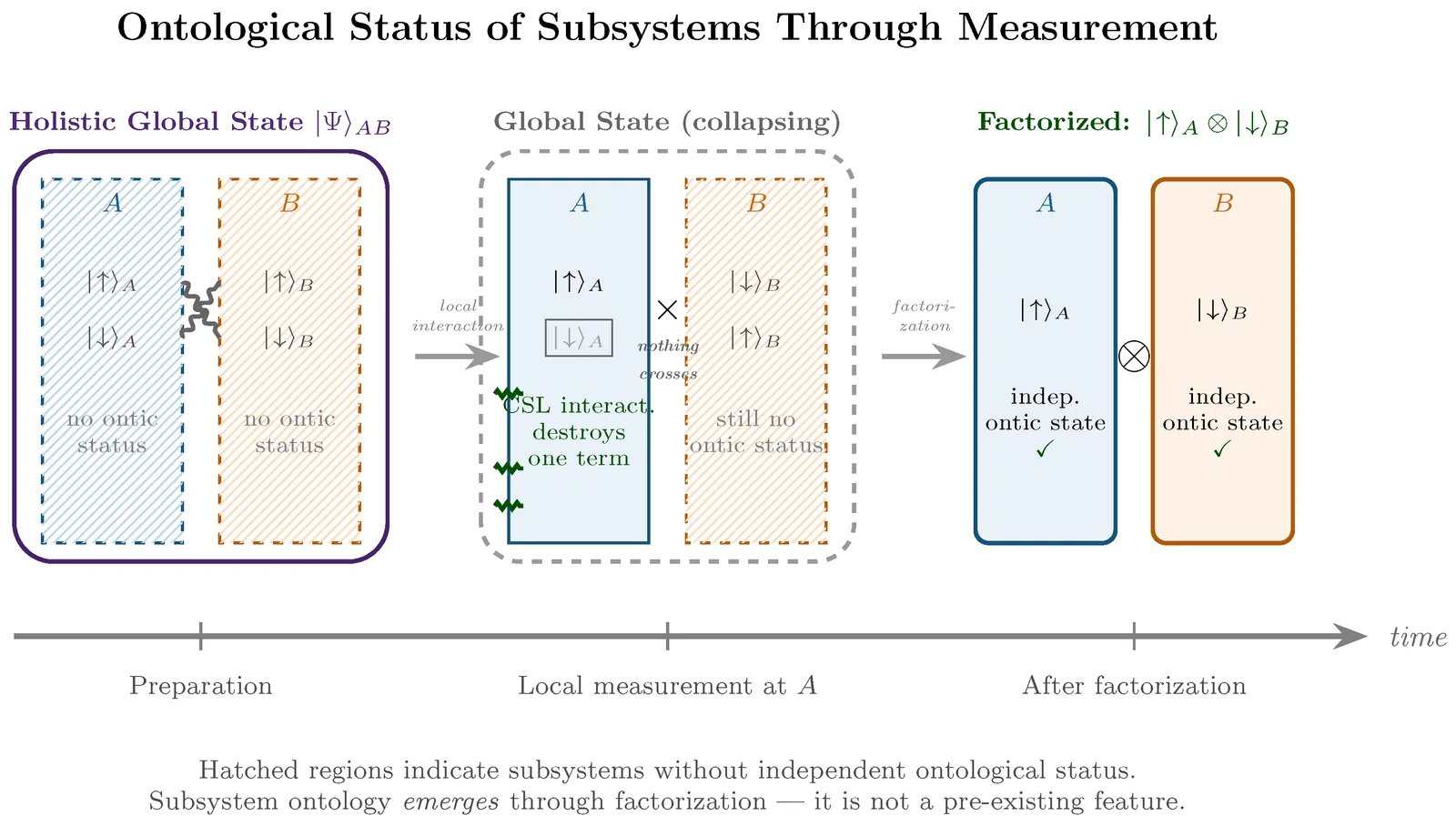
Ontological status of subsystems through measurement. Hatched regions indicate subsystems without independent ontological status. At preparation (**left**), the global wavefunction ${|\Psi \rangle }_{AB}$ is the sole ontic object; subsystems A and B have no independent states. During local measurement (**center**), CSL interaction at A destroys one component while B remains untouched. After factorization (**right**), both subsystems acquire independent ontic states for the first time. Subsystem ontology *emerges* through factorization—it is not a pre-existing feature.

This is analogous to asking: “When did the individual drop become a separate entity from the ocean?” The question assumes the drop always existed as an independent thing. But the drop only becomes a meaningful individual entity when it separates from the ocean. Similarly, B’s “state” only becomes a meaningful ontic entity when the global wavefunction factorizes. (We note that the analogy is imperfect: a water drop has a definite spatial boundary even before separation, whereas in the quantum case the subsystem has no independent properties whatsoever prior to factorization. The analogy illustrates the emergence of individuality, not the degree of prior independence.) The ontological timeline of the subsystems through this process is summarized in Figure 2.

## 6. Bell’s Theorem, Contextuality, and the GHZ Result

### 6.1. What Bell’s Theorem Assumes

We state Bell’s theorem in the form appropriate to its full generality. In Bell’s 1964 argument [2], determinism was not an independent premise: it was *derived*, via the EPR reality criterion, from perfect anticorrelations together with locality. The later stochastic formulations—Clauser–Horne [18], Bell’s own local-causality formulation, and Fine’s theorem [19] (see Norsen [12] for a careful analysis)—require no determinism at all. The operative assumption is factorizability (L3 of Section 2.3):

(9) P(a,b|x,y,λ)=P(a|x,λ)P(b|y,λ),

together with measurement-setting independence of λ. Stochastic local response functions satisfying this condition obey the Bell inequalities, and any local randomness can be absorbed into an enlarged λ without changing the empirical content. Quantum mechanics violates the inequalities; therefore factorizability fails—and it fails in *every* ontological reading of quantum mechanics, ours included. The interpretive question is not *whether* (L3) fails but *where*: in the parameter-independence factor (a dynamical influence of the distant setting) or in the outcome-independence factor (a correlation between outcomes unscreened by λ).

### 6.2. Our Response: Locating the Failure of Factorizability in Outcome Independence

Our answer locates the failure entirely in outcome independence. With λ=|Ψ〉—the natural and canonical choice in a ψ-ontic framework—parameter independence holds exactly (Theorem 4 and Section 6.7): neither wing’s statistics respond to the distant setting, reflecting the strict locality of the dynamics (L2). Outcome independence fails: conditioning on Alice’s outcome changes the probabilities for Bob’s, and no enlargement of λ can screen this off, because the correlation is carried by the nonseparable structure of |Ψ〉 itself. This is a genuine violation of Bell local causality—we do not dispute Bell’s theorem—but it is a violation of a very particular kind: one located in the ontology (what the world’s state is like) rather than in the dynamics (what influences what). The remainder of this section develops the independent reasons for also rejecting pre-assigned measurement values.

Our ψ-ontic ontology additionally rejects outcome determinism: observables do not possess definite values prior to measurement. For entangled systems, the question “what is the spin of particle B along axis $\hat{n}$?” is ill-posed—not because the answer is unknown, but because B does not have an independent state from which a definite spin value could be derived.

This rejection is not ad hoc; it is supported by independent no-go theorems. Moreover, the original route to outcome determinism—the EPR inference from perfect correlations via the reality criterion [1]—already presupposes that each subsystem possesses an independent real state, since only then can Alice’s measurement on A reveal a property that “belongs” to B. That presupposition is precisely what our holistic ontology denies: for a non-factorizable global state there is no subsystem state at B to which a definite pre-existing value could be attributed, so the EPR inference from correlation to determinism does not get off the ground.

### 6.3. The Kochen–Specker Theorem

The Kochen–Specker theorem [26] demonstrates that in Hilbert space dimension d≥3, it is impossible to assign definite values to all observables simultaneously in a manner consistent with the functional relations between commuting observables (see also [27] for related arguments). The conceptual roots of this contextuality trace back to early analyses of the measurement problem [28]. Formally:

**Theorem** **1** (Kochen–Specker)**.** *There exists no valuation function v:O→R from the set of self-adjoint operators on a Hilbert space H with dim(H)≥3 to the reals satisfying:*

*(KS1) $v\left(\hat{A}\right)\in \sigma \left(\hat{A}\right)$ for all observables $\hat{A}$ (values are eigenvalues), and (KS2) $v\left(f\left(\hat{A}\right)\right)=f\left(v\left(\hat{A}\right)\right)$ for all observables $\hat{A}$ and functions f (functional composition is preserved).*

This is a statement about the structure of quantum mechanics itself, independent of entanglement or locality. It establishes that the assignment of definite pre-existing values to all observables is mathematically inconsistent—not merely empirically falsified, but logically impossible.

### 6.4. The GHZ Theorem: A Direct Refutation of Outcome Determinism

The GHZ theorem [29,30] provides a dramatic strengthening of the Kochen–Specker result, establishing a direct logical contradiction—not a statistical violation—between outcome determinism and quantum predictions. We present the argument in detail, as it is central to our framework.

**The GHZ State.** Consider three spin-$\frac{1}{2}$ particles in the GHZ state:
  (10) $$|\mathrm{GHZ}\rangle =\frac{1}{\sqrt{2}}\left(|↑↑↑\rangle -|↓↓↓\rangle \right).$$
**Quantum predictions.** The following four mutually commuting observables have definite eigenvalues on |GHZ〉:
  (11) $${\hat{O}}_{1}=\sigma_{x}^{\left(1\right)}\sigma_{y}^{\left(2\right)}\sigma_{y}^{\left(3\right)},\;{\hat{O}}_{2}=\sigma_{y}^{\left(1\right)}\sigma_{x}^{\left(2\right)}\sigma_{y}^{\left(3\right)},\;{\hat{O}}_{3}=\sigma_{y}^{\left(1\right)}\sigma_{y}^{\left(2\right)}\sigma_{x}^{\left(3\right)},\;{\hat{O}}_{4}=\sigma_{x}^{\left(1\right)}\sigma_{x}^{\left(2\right)}\sigma_{x}^{\left(3\right)}.$$
  Direct calculation yields:
  (12) $${\hat{O}}_{1}|\mathrm{GHZ}\rangle =+|\mathrm{GHZ}\rangle ,\;{\hat{O}}_{2}|\mathrm{GHZ}\rangle =+|\mathrm{GHZ}\rangle ,\;{\hat{O}}_{3}|\mathrm{GHZ}\rangle =+|\mathrm{GHZ}\rangle ,$$
  (13) $${\hat{O}}_{4}|\mathrm{GHZ}\rangle =-|\mathrm{GHZ}\rangle .$$
**The contradiction from outcome determinism.** Suppose, contrary to our ontology, that each particle possesses definite pre-existing values for both $\sigma_{x}$ and $\sigma_{y}$. Denote these values as $X_{i}=v\left(\sigma_{x}^{\left(i\right)}\right)=\pm 1$ and $Y_{i}=v\left(\sigma_{y}^{\left(i\right)}\right)=\pm 1$ for i=1,2,3.

From the eigenvalue equations above with the functional composition rule:

(14) $$\begin{array}{cc} X_{1}Y_{2}Y_{3} & =+1\;(\mathrm{from}\;{\hat{O}}_{1}), \end{array}$$

(15) $$\begin{array}{cc} Y_{1}X_{2}Y_{3} & =+1\;(\mathrm{from}\;{\hat{O}}_{2}), \end{array}$$

(16) $$\begin{array}{cc} Y_{1}Y_{2}X_{3} & =+1\;(\mathrm{from}\;{\hat{O}}_{3}). \end{array}$$

Multiplying all three equations:

(17) $$\left(X_{1}Y_{2}Y_{3}\right)\left(Y_{1}X_{2}Y_{3}\right)\left(Y_{1}Y_{2}X_{3}\right)=\left(+1\right)\left(+1\right)\left(+1\right)=+1.$$

Since $Y_{i}^{2}=1$ for all *i*, this simplifies to:

(18) $$X_{1}X_{2}X_{3}\cdot Y_{1}^{2}Y_{2}^{2}Y_{3}^{2}=X_{1}X_{2}X_{3}=+1.$$

But the fourth eigenvalue equation requires:

(19) $$X_{1}X_{2}X_{3}=-1\;(\mathrm{from}\;{\hat{O}}_{4}).$$

This is a direct logical contradiction: +1≠−1. No assignment of definite values to the observables can reproduce all four quantum predictions simultaneously. The assumption of outcome determinism is refuted—not statistically, but with logical certainty.

**Significance for our framework—stated with care.** The displayed derivation excludes exactly one thing: *noncontextual* value assignments—assignments of context-independent values $X_{i},Y_{i}$ used identically across the four joint measurement contexts. It does not exclude deterministic *contextual* theories: Bohmian mechanics, in which the outcome of each measurement depends on the full experimental arrangement, reproduces every GHZ prediction, at the price of dynamical nonlocality. Accordingly, GHZ does not refute “outcome determinism alone, independently of locality and contextuality.” What GHZ (with Kochen–Specker) does establish is decisive for our purposes: the EPR route from perfect correlations to pre-existing values is blocked, because the values so inferred would have to be noncontextual, and no such assignment exists. Our framework embraces the resulting contextuality of measurement outcomes—outcomes are generated in the measurement process, relative to the full context—while keeping the dynamics local; it is contextual about *values* and local about *influences*.

### 6.5. Observable Incompatibility and the Landau Identity

Beyond the GHZ argument, the violation of Bell inequalities can be understood directly as a consequence of the incompatibility of quantum observables, without any reference to nonlocality [6,15]. Using the CHSH operator $\hat{S}=\hat{A}\hat{B}-\hat{A}{\hat{B}}^{\prime }+{\hat{A}}^{\prime }\hat{B}+{\hat{A}}^{\prime }{\hat{B}}^{\prime }$ and the identity ${\hat{A}}^{2}={\hat{A}}^{\prime 2}={\hat{B}}^{2}={\hat{B}}^{\prime 2}=I$, Landau [31] derived:

(20) $${\hat{S}}^{2}=4I+\left[\hat{A},{\hat{A}}^{\prime }\right]\left[\hat{B},{\hat{B}}^{\prime }\right].$$

When observables within each subsystem commute ($[\hat{A},{\hat{A}}^{\prime }]=0$ or $[\hat{B},{\hat{B}}^{\prime }]=0$), we recover $\langle {\hat{S}}^{2}\rangle =4$ and hence |S|≤2—the classical Bell bound. In EPR–Bell scenarios, however, the local observables are incompatible ($[\hat{A},{\hat{A}}^{\prime }]\neq 0$ and $[\hat{B},{\hat{B}}^{\prime }]\neq 0$), and the additional term permits $\left|S\right|\leq 2\sqrt{2}$ (the Tsirelson bound). Thus, Bell violations arise from the noncommutativity of local observables, not from nonlocal influences between the subsystems.

### 6.6. Contextuality and the Origin of Correlations

Our framework embraces contextuality: the outcome of a measurement depends on the measurement context, not on pre-existing properties of the system. For entangled systems, the “context” includes the global wavefunction structure, which determines the correlations between possible measurement outcomes at A and B.

Crucially, contextuality does not require nonlocal dynamics. The correlations between A and B are established at preparation—they are encoded in the non-factorizability of the global wavefunction. Measurement at A does not “send” information to B; it locally modifies the global wavefunction, causing factorization. The correlations that were latent in the entangled state become manifest as definite correlated outcomes only when both measurements have been performed and results compared.

To make this precise: consider Alice choosing to measure either $\sigma_{z}$ or $\sigma_{x}$ on her particle. In the standard account, Alice’s choice “steers” Bob’s state into different ensembles (eigenstates of $\sigma_{z}$ or $\sigma_{x}$). In our account, Alice’s choice determines which local wavefunction component at A is destroyed by the collapse dynamics. The resulting factorized state has different structure depending on Alice’s basis choice, but at no point does any physical influence propagate to B. The correlations emerge from the pre-existing global structure when results are compared.

### 6.7. Addressing Stronger Bell-Type Results

We acknowledge that there exist Bell-type results with weakened assumptions [16,17]. In particular, some formulations replace full outcome determinism with weaker conditions such as:

(OI) **Outcome independence:** The probability of an outcome at A, given the hidden variable λ and the measurement settings at both stations, does not depend on the outcome at B.
(PI) **Parameter independence:** The probability of an outcome at A, given λ and the outcome at B, does not depend on the measurement setting at B.

In a ψ-ontic framework the Jarrett–Shimony decomposition applies directly and canonically: the complete ontic state is λ=|Ψ〉, and no “hypothetical” imposition is needed. The resulting description satisfies parameter independence (PI) exactly and violates outcome independence (OI): the global wavefunction encodes correlations between outcomes that cannot be screened off, precisely because λ itself is nonseparable. We state plainly what this means: Bell local causality—condition (L3) of Section 2.3—fails in our framework, as it must in any empirically adequate account. Our thesis is about the *character* of that failure: it is a failure of outcome independence carried by the structure of the ontic state, with parameter independence and dynamical locality (L2) fully intact. Calling this “nonseparability rather than nonlocality” is an interpretive identification—one we defend, with Howard [8] and Jarrett [16], on the ground that the (L1)/(L2)-preserving, uncontrollable, frame-independent character of outcome dependence is naturally read as a feature of what the world is like rather than of what influences what—but it does not, and is not meant to, contest Bell’s theorem.

Parameter independence (PI) would be preserved in our framework: since measurement at A involves only local dynamics, the statistics at A cannot depend on Bob’s distant measurement setting. This is precisely the content of the no-signaling theorem.

### 6.8. No-Signaling as Ontological Constraint

The no-signaling theorem states that the marginal measurement statistics at B are independent of what measurement (if any) is performed at A. In our framework, this has a natural ontological explanation: since nothing physical happens at B when A is measured, there is nothing for B’s local measurement statistics to depend on. No-signaling is not a fortuitous cancellation; it is a direct consequence of the locality of measurement dynamics.

Formally, for any local observable ${\hat{O}}_{B}$ acting on B’s Hilbert space:

(21) $$\langle {\hat{O}}_{B}\rangle =\mathrm{Tr}\left(\rho_{B}{\hat{O}}_{B}\right)=\mathrm{Tr}\left({\mathrm{Tr}}_{A}(|\Psi \rangle \langle \Psi |)\;{\hat{O}}_{B}\right).$$

This trace depends only on the global state |Ψ〉 and is independent of any measurement performed on A. In our ontology, this mathematical fact reflects a physical truth: A’s local dynamics do not propagate to B.

### 6.9. Quantum Steering

A further challenge comes from the quantum steering framework [32], which provides an operational criterion for “what changes at B when A is measured” without presupposing outcome determinism. Steering inequalities are violated by quantum mechanics, and their violation is sometimes interpreted as evidence that Alice’s measurement choice genuinely affects Bob’s state.

To make the operative distinction fully explicit, we adopt the standard instrument formalism [33]. For a local measurement on A with Kraus operators $\left\{M_{a}\right\}$, the state *conditioned* on outcome *a* (selective) is

(22) $$\rho_{AB|a}\;=\;\frac{\left(M_{a}⊗I_{B}\right)\;\rho_{AB}\;\left(M_{a}^{†}⊗I_{B}\right)}{p_{a}},\;p_{a}=\mathrm{Tr}\left[\left(M_{a}^{†}M_{a}⊗I_{B}\right)\;\rho_{AB}\right],$$

so Bob’s conditional state $\rho_{B|a}={\mathrm{Tr}}_{A}\;\rho_{AB|a}$ is outcome-dependent and may be pure; whereas if the outcome is not conditioned upon (nonselective), $\rho_{B}^{\prime }=\sum_{a}p_{a}\;\rho_{B|a}=\rho_{B}$—the content of Theorem 4. These two descriptions answer different questions and are never interchangeable. Theorem 4 establishes the nonselective statement only: it does not, by itself, show that a selected trajectory carries no ontological consequence associated with B. That further claim is exactly where our interpretive postulate does its work: on our reading, the outcome dependence of $\rho_{B|a}$ is conditioning on information about the nonseparable global state (the OI violation of Section 6.7), not the record of a physical change at B. The operational difference between the conditioned and unconditioned descriptions of B is quantified, from an information-theoretic direction, by the quantum-memory-assisted entropic uncertainty literature, in which conditioning on measurements of one subsystem tightens the predictive bounds for its entangled partner while leaving the partner’s unconditioned marginal untouched [34,35,36,37]; a concrete bipartite realization of this quantum-memory effect—measurements on the spin subsystem of a spin-orbit-coupled double quantum dot reducing the uncertainty of its orbital partner—is analyzed in [38].

Our framework responds as follows: steering describes the fact that Alice, by choosing her measurement basis, can remotely prepare different *ensembles* for Bob’s subsystem. In the standard account, this is taken as evidence of nonlocal influence. In our account, Alice’s measurement choice determines which local wavefunction component at A is destroyed by the collapse dynamics. The different ensembles that Bob would observe (upon learning Alice’s result) reflect the different ways the global wavefunction can factorize—they are consequences of the pre-existing holistic structure, not of any signal from A to B. Crucially, Bob cannot detect any difference in his local statistics without access to Alice’s results (no-signaling), which is precisely what our ontology predicts: nothing physical changed at B.

## 7. Formal Framework: Definitions and Theorems

We now formalize the key concepts of our framework in precise mathematical terms.

**Definition** **1** (Global Ontic State)**.** *For a composite quantum system with Hilbert space $H=H_{A}⊗H_{B}$, the **global ontic state** is the normalized vector |Ψ〉∈H of the total *isolated* system. In our monist framework the fundamental state is always pure; density operators arise only as improper reductions of a larger pure state (system plus apparatus plus environment) and inherit no independent ontological status. This is the sole bearer of ontological status.*

**Remark (mixed-state vocabulary).**  When density operators appear below, the standard distinctions must be kept sharp: a *product* state $\rho_{AB}=\rho_{A}⊗\rho_{B}$; a *separable* state $\rho_{AB}=\sum_{i}p_{i}\;\rho_{A}^{\left(i\right)}⊗\rho_{B}^{\left(i\right)}$, which may carry classical correlations without being a product; and a *classically correlated* decohered state, which is separable but generally not a product. A product state may have mixed reduced states (e.g., $\rho_{A}⊗I_{B}/2$), so purity of $\rho_{B}$ is *not* a necessary condition for B to possess an independent state once mixed global states are admitted—and a maximal CHSH value of 2 does not imply a product state. For this reason, all factorization-based ontological claims in this paper (Definition 3, Theorems 2 and 3) are made at the level of the *pure* state of the total isolated system, where they are exact; statements about decohered subsystems are effective statements within branches of that global pure state (Section 8).

**Definition** **2** (Factorizability)**.** *A pure global state $|\Psi \rangle \in H_{A}⊗H_{B}$ is **factorizable** (separable) if and only if there exist $|\psi_{A}\rangle \in H_{A}$ and $|\psi_{B}\rangle \in H_{B}$ such that $\left|\Psi \rangle =\right|\psi_{A}\rangle ⊗|\psi_{B}\rangle$. A state that is not factorizable is **entangled***.

**Definition** **3** (Subsystem Ontic State)**.** *A subsystem B possesses an **independent ontic state** if and only if the global state is factorizable. In this case, the subsystem ontic state is the factor $|\psi_{B}\rangle$. For entangled (non-factorizable) states, subsystem B does not possess an independent ontic state.*

**Definition** **4** (Local Dynamical Process)**.** *A dynamical process is **local at A** if every operator through which it acts—Hamiltonians, Kraus operators, collapse operators, noise couplings—has the form $X_{A}⊗I_{B}$ with $X_{A}$ supported on $H_{A}$ (and its local environment). This single support condition is applied at three levels, which must be distinguished: (i) the* selective, unnormalized *level, where conditioning on an outcome a is the completely positive, trace-nonincreasing operation $\rho \mapsto \left(M_{a}⊗I_{B}\right)\rho \left(M_{a}^{†}⊗I_{B}\right)$; (ii) the *selective, normalized* level of individual trajectories, where division by $p_{a}$ (or the normalization of a stochastic collapse equation) makes the map nonlinear—locality here means that every operator entering the trajectory equation is of the form $X_{A}⊗I_{B}$; and (iii) the *nonselective ensemble* level, $E_{A}⊗I_{B}$, which is linear and trace-preserving. Level (iii) supports the no-signaling statement (Theorem 4); levels (i)–(ii) describe single realizations. The locality claimed in this paper is the operator-support condition, uniformly across all three levels; it is condition (L2) of Section 2.3, and it neither implies nor is meant to imply Bell local causality (L3).*

**Theorem** **2** (Entanglement–Separability Equivalence)**.** *A pure bipartite state $|\Psi \rangle \in H_{A}⊗H_{B}$ is factorizable if and only if $\rho_{B}={\mathrm{Tr}}_{A}(|\Psi \rangle \langle \Psi |)$ is a pure state.*

**Proof.**  (⇒) If $|\Psi \rangle =|\psi_{A}\rangle ⊗|\psi_{B}\rangle$, then $\rho_{B}={\mathrm{Tr}}_{A}(|\psi_{A}\rangle \langle \psi_{A}|⊗|\psi_{B}\rangle \langle \psi_{B}|)=|\psi_{B}\rangle \langle \psi_{B}|$, which is pure.

(⇐) If $\rho_{B}$ is pure, then by the Schmidt decomposition $|\Psi \rangle =\sum_{i}\lambda_{i}|a_{i}\rangle ⊗|b_{i}\rangle$ with Schmidt coefficients $\lambda_{i}\geq 0$ (non-negative by convention, since phases are absorbed into the basis vectors) and $\sum_{i}\lambda_{i}^{2}=1$, we have $\rho_{B}=\sum_{i}\lambda_{i}^{2}|b_{i}\rangle \langle b_{i}|$. Purity of $\rho_{B}$ requires $\mathrm{Tr}\left(\rho_{B}^{2}\right)=\sum_{i}\lambda_{i}^{4}=1$. Combined with $\sum_{i}\lambda_{i}^{2}=1$, this implies that exactly one $\lambda_{i}=1$ and the rest vanish. Hence $\left|\Psi \rangle =\right|a_{j}\rangle ⊗|b_{j}\rangle$ is factorizable. □

**Ontological interpretation:** Subsystem B possesses an independent ontic state if and only if $\rho_{B}$ is pure. The mixed-to-pure transition of $\rho_{B}$ upon measurement of A marks the emergence of subsystem ontology, not a nonlocal disturbance.

**Theorem** **3** (Local Collapse Induces Factorization)**.** *Let $|\Psi \rangle =\sum_{i}c_{i}|\alpha_{i}\rangle_{A}⊗{|\beta_{i}\rangle }_{B}$ be an entangled state with at least two nonzero coefficients $c_{i}$, where ${\{|\alpha_{i}\rangle }_{A}\}$ are orthogonal. If a local dynamical process at A drives all coefficients to zero except $c_{k}$ (i.e., $c_{i}\to \delta_{ik}$ for some k), then the resulting state is factorizable: $|\Psi^{\prime }\rangle =|\alpha_{k}\rangle_{A}⊗{|\beta_{k}\rangle }_{B}$.*

**Proof.**  By hypothesis, the local process at A maps $c_{i}\to \delta_{ik}$. After renormalization: $|\Psi^{\prime }\rangle =|\alpha_{k}\rangle_{A}⊗{|\beta_{k}\rangle }_{B}$, which is manifestly factorizable. The process modifies only the coefficients associated with A’s orthogonal components ${\{|\alpha_{i}\rangle }_{A}\}$ and does not alter the states ${\{|\beta_{i}\rangle }_{B}\}$. □

**Ontological interpretation:** A local process at A—one that acts only on A’s wavefunction components—suffices to factorize the global state. No dynamics at B are required. The factorization is a consequence of local collapse, not nonlocal influence. We note that this theorem captures the *structural* consequence of local collapse; the substantive physical question of whether a specific collapse model (such as CSL) constitutes a genuinely local process—in the terms of Section 2.3, and particularly given known tensions with Lorentz covariance—requires separate argument and is discussed in Section 10.6 (a) Relativistic formulation part.

**Theorem** **4** (No-Signaling from Local Collapse)**.** *Let $|\Psi \rangle \in H_{A}⊗H_{B}$ and let $\left\{P_{a}^{A}\right\}$ be a complete set of orthogonal projectors implementing a measurement on A, with $P_{a}^{A}P_{a^{\prime }}^{A}=\delta_{aa^{\prime }}P_{a}^{A}$ and $\sum_{a}P_{a}^{A}=I_{A}$. The reduced density matrix of B, averaged over all possible measurement outcomes at A, is unchanged:*

(23) $$\sum_{a}{\mathrm{Tr}}_{A}\left[\left(P_{a}^{A}⊗I_{B}\right)\left|\Psi \rangle \langle \Psi \right|\left(P_{a}^{A}⊗I_{B}\right)\right]={\mathrm{Tr}}_{A}\left(|\Psi \rangle \langle \Psi |\right)=\rho_{B}\;.$$

**Proof.**  Using the idempotency of each projector, $\left(P_{a}^{A}⊗I_{B}\right)\left(P_{a}^{A}⊗I_{B}\right)=P_{a}^{A}⊗I_{B}$, and the cyclic property of the partial trace over A, we move one projector past |Ψ〉〈Ψ|:

(24) $$\begin{array}{cc} \sum_{a}{\mathrm{Tr}}_{A}\left[\left(P_{a}^{A}⊗I_{B}\right)\left|\Psi \rangle \langle \Psi \right|\left(P_{a}^{A}⊗I_{B}\right)\right] & =\sum_{a}{\mathrm{Tr}}_{A}\left[\left(P_{a}^{A}⊗I_{B}\right)\left(P_{a}^{A}⊗I_{B}\right)\;|\Psi \rangle \langle \Psi |\right]\\ \; & =\sum_{a}{\mathrm{Tr}}_{A}\left[\left(P_{a}^{A}⊗I_{B}\right)\;|\Psi \rangle \langle \Psi |\right]\\ \; & ={\mathrm{Tr}}_{A}\left[\left(\sum_{a}P_{a}^{A}\right)⊗I_{B}\cdot \left|\Psi \rangle \langle \Psi \right|\right]\\ \; & ={\mathrm{Tr}}_{A}\left(|\Psi \rangle \langle \Psi |\right)=\rho_{B}\;, \end{array}$$

where completeness, $\sum_{a}P_{a}^{A}=I_{A}$, was used in the last step. □

**Ontological interpretation—scope stated precisely:** Before Alice communicates her result to Bob, Bob’s unconditioned local statistics are completely unaffected by Alice’s measurement. This is not a fine-tuned cancellation but a direct consequence of the mathematical structure: local operations at A cannot alter the partial trace over A. We are explicit about what this theorem does and does not establish. It establishes the statistical no-signaling property (L1) of nonselective local operations. It does not, by itself, establish that a selected collapse trajectory is local in the Bell-causal sense (L3)—nothing can, since (L3) fails—nor even that the selected trajectory carries no ontological association with B; that interpretive step is supplied by the holism postulate of Section 3, under which the outcome dependence of Bob’s *conditional* description is conditioning on the nonseparable global state rather than the record of a change at B. The theorem’s role in our argument is thus supporting, not load-bearing: it certifies (L1), while (L2) is certified by the operator-support structure of the dynamics (Definition 4) and the (L3)-failure is embraced and relocated into the ontology (Section 6.7).

## 8. Decoherence and the Emergence of Separability

### 8.1. Decoherence as Factorization

Environmental decoherence [25,39,40] provides a universal mechanism for the emergence of separability. When a system S interacts with its environment E, the combined state evolves from a product state to an entangled state:

(25) $$|\psi_{S}\rangle ⊗|E_{0}\rangle \;\to \;\sum_{i}c_{i}|s_{i}\rangle ⊗|E_{i}\rangle ,$$

where $\left\{|s_{i}\rangle \right\}$ are the pointer states selected by the interaction Hamiltonian, and $\left\{|E_{i}\rangle \right\}$ are approximately orthogonal environment states. The reduced density matrix of S becomes approximately diagonal in the pointer basis:

(26) $$\rho_{S}\approx \sum_{i}|c_{i}{|}^{2}|s_{i}\rangle \langle s_{i}|.$$

In our framework, this process has a precise ontological interpretation: decoherence progressively factorizes the global wavefunction into branches that are effectively independent. Each branch $|s_{i}\rangle ⊗|E_{i}\rangle$ represents a factorized subsector of the global state. Within each branch, the system S has an effective independent state—the pointer state $|s_{i}\rangle$.

### 8.2. The Ubiquity of Decoherence

Decoherence is extraordinarily efficient. For macroscopic objects at room temperature, decoherence timescales are of order $10^{-26}$ s—far shorter than any experimentally resolvable timescale [39]. Even for mesoscopic systems (molecules, nanoparticles), decoherence occurs on timescales of nanoseconds to microseconds.

The practical consequence is that entanglement between spatially separated macroscopic systems is extremely fragile. Two particles prepared in an entangled state will rapidly decohere—their global wavefunction will factorize—unless they are carefully isolated from the environment (as in laboratory EPR experiments). In the natural world, almost all systems are effectively separable because decoherence has factorized the global wavefunction.

A remarkable feature of this process, established by the corrected analysis in [5], is that the maximal Bell–CHSH value evolves as $S_{\max}\left(t\right)=2\sqrt{1+{\left|z\left(t\right)\right|}^{2}}$: it decays smoothly from the Tsirelson bound $2\sqrt{2}$ to the classical bound 2 as decoherence completes, consuming exactly the coherence on which CHSH violation depends, while the outcome probabilities in the measurement basis remain exactly preserved. This means that the quantum correlations responsible for Bell violations are structural properties of the initial entangled state—encoded in the non-factorizability of the global wavefunction—and their decay is a purely local effect of environmental entanglement, not the action of any nonlocal influence. Decoherence destroys local coherence and drives factorization, and the correlations that emerge when measurement results are compared reflect what remains of this pre-existing holistic structure. This is precisely what our ontology predicts: the correlations are holistic features of the global wavefunction, not products of nonlocal signaling.

The decay of $S_{\max}\left(t\right)$ from $2\sqrt{2}$ to 2 thus admits a direct ontological reading. While |z(t)|>0, the global state remains non-factorizable: A and B possess no independent ontic states, and the residual violation $S_{\max}>2$ is the quantitative signature of that holism—correlations that cannot be decomposed into pre-existing subsystem properties precisely because the subsystems do not yet exist as independent entities. As decoherence completes (|z(t)|→0), the global wavefunction factorizes into pointer-state branches, independent subsystem states emerge from it for the first time, and the maximal correlation settles at the classical bound 2—the highest value a fully factorized, subsystem-based description can support. The trajectory from $S_{\max}=2\sqrt{2}$ to $S_{\max}=2$ is, in this sense, the dynamical record of subsystem ontology emerging out of the holistic wavefunction.

### 8.3. Classical Separability as Emergent

This provides a complete account of why the classical world appears separable despite our holistic quantum ontology:

(a) **Fundamental level:** The universal wavefunction $\Psi (x_{1},x_{2},\ldots ,x_{N})$ is the sole ontic object. It lives in configuration space and is generically non-factorizable.
(b) **Decoherence:** Interactions with the environment rapidly factorize the wavefunction into effectively independent branches, each of which describes subsystems with definite pointer states.
(c) **Classical appearance:** The factorized branches correspond to the classical world of independent objects with definite properties. Separability is not fundamental but emergent.
(d) **Entanglement as exception:** Laboratory entanglement experiments succeed precisely because the particles are carefully isolated from decoherence. The EPR scenario is the exception, not the rule.

## 9. Relation to Existing Frameworks

### 9.1. Howard’s Separability–Locality Distinction

Don Howard [8] drew a fundamental distinction between separability and locality in the context of the EPR argument. *Separability* holds that spatially separated systems possess independent real states. *Locality* holds that the real state of a system is not affected by events at spacelike separation. Howard argued that Einstein’s actual concern was separability, not locality, and that quantum mechanics violates separability while potentially preserving locality. Our framework aligns closely with Howard’s analysis: we deny separability (for entangled systems) while maintaining locality (measurement dynamics are local).

### 9.2. Teller’s Relational Holism

Paul Teller [9] proposed relational holism: entangled systems possess irreducible relational properties that are not determined by the intrinsic properties of the parts (see also Esfeld [41] for related discussion). Our ψ-ontic holism goes further: for entangled systems, the parts do not possess intrinsic properties at all. The global wavefunction is the sole bearer of properties. Teller’s relational properties are, in our view, properties of the global wavefunction that cannot be decomposed into subsystem contributions.

### 9.3. Maudlin’s Nonlocality

Tim Maudlin [11] has argued forcefully that quantum mechanics is genuinely nonlocal, and that Bell’s theorem establishes nonlocal causation, not merely nonlocal correlation. Our disagreement with Maudlin is now precisely locatable in the terms of Section 2.3. We agree that Bell local causality (L3) fails—this much is theorem, and we do not dispute it. Maudlin reads the failure as nonlocal *causation*; we read it as nonseparability of the ontic state: the violation is confined to outcome independence, is uncontrollable, selects no frame, and leaves parameter independence and the operator-support locality of the dynamics (L2) fully intact. On our account the correlations are encoded in the global wavefunction from preparation and require no causal transmission across spacelike separation. Which reading is correct is an interpretive question—Jarrett [16] and Howard [8] laid out the case that outcome dependence without parameter dependence is naturally a feature of what the world is like rather than of what influences what—and it is that case, not a rejection of Bell’s theorem, on which our disagreement with Maudlin rests.

### 9.4. CSL and Objective Collapse Models

**Figure 3:**
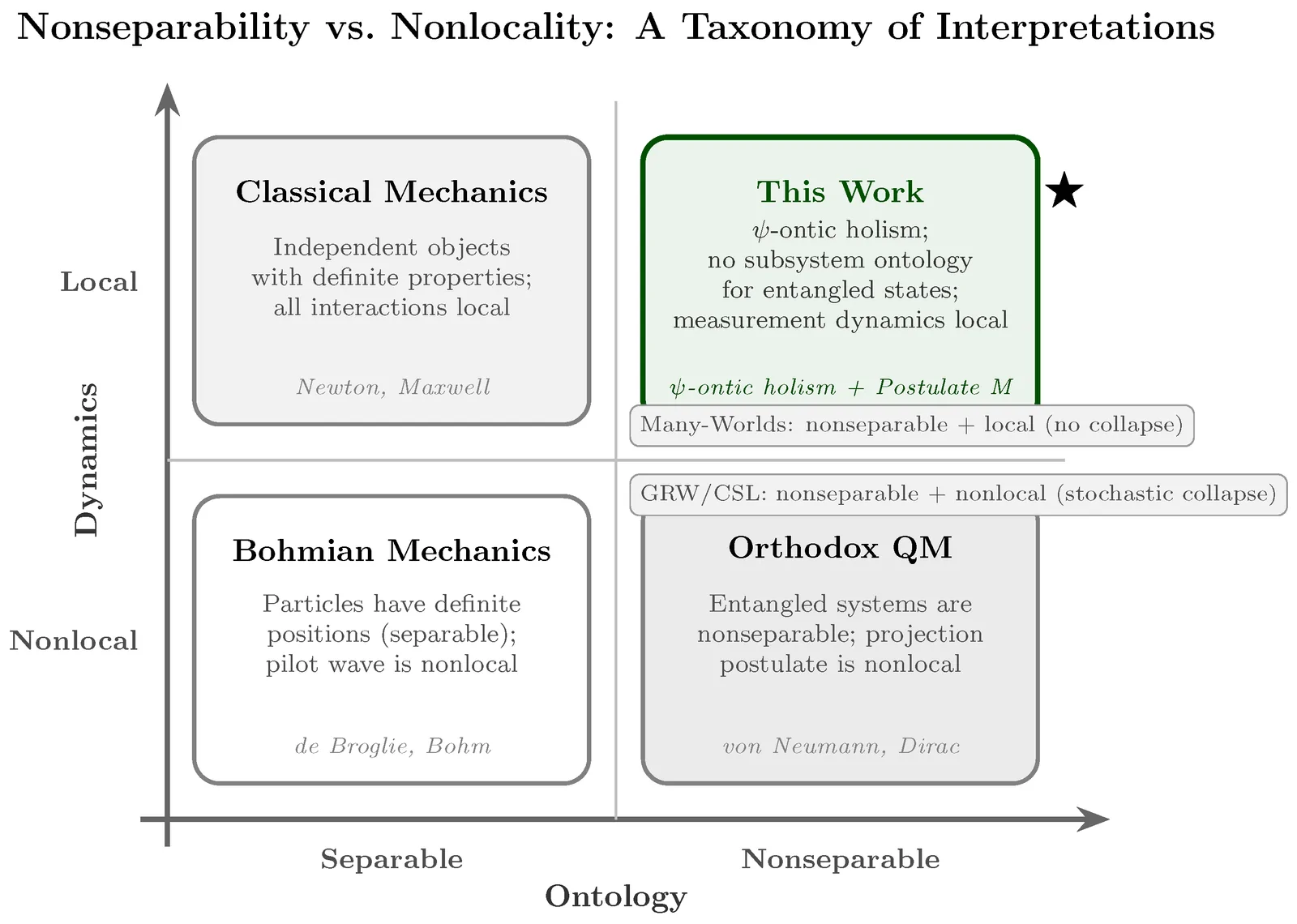
Taxonomy of interpretations classified by ontology (separable vs. nonseparable) and dynamics (local vs. nonlocal). Classical mechanics occupies the separable–local quadrant; Bohmian mechanics is separable–nonlocal; orthodox quantum mechanics is nonseparable–nonlocal. Our framework (ψ-ontic holism with Postulate M) occupies the nonseparable–local quadrant (starred): the ontology is holistic, but measurement dynamics are local. GRW/CSL appears in the nonseparable–nonlocal cell *as standardly interpreted*, i.e., with the collapse of an entangled state read as an instantaneous global event; the re-reading developed in this paper and in Postulate M [5]—collapse as locally triggered branch selection—relocates the same dynamical models to the nonseparable–local cell. The figure classifies interpretations, not equations: the placement encodes the reading of the collapse, and the two placements of CSL-type dynamics are not in contradiction.

The GRW [42] and CSL [21,22] programs provide objective, dynamical collapse mechanisms that modify the Schrödinger equation. As standardly interpreted, these models prevent superluminal signaling at the statistical level while their individual collapse histories for entangled states are nonlocal in Bell’s sense—which is why Figure 3 places standard GRW/CSL in the nonseparable–nonlocal cell. What we take from CSL is its *dynamical* content—operators and noise with strictly local support—while re-reading the realized outcome as locally triggered branch selection on the nonseparable state, per Postulate M [5]; on that reading, and only on that reading, the same equations occupy the nonseparable–local cell. Our use of CSL is as an illustration of local collapse dynamics, not as a commitment to the specific CSL mechanism. We agree with the CSL program that collapse is a physical process, but our emphasis is different: CSL focuses on solving the measurement problem (why do we see definite outcomes?), while we focus on the locality of collapse (why is there no nonlocal influence?).

### 9.5. Everettian Quantum Mechanics

The many-worlds interpretation [14] shares our commitment to the global wavefunction as the sole ontic object and agrees that no nonlocal collapse occurs. However, it achieves this by denying collapse entirely: all branches persist, and the appearance of definite outcomes is explained by decoherence and branch-relative experience. Our framework is compatible with an Everettian reading (in which “factorization” refers to effective decoherence-induced branching rather than physical collapse), but also with objective collapse readings. The holistic ontology and the locality argument are independent of the single-outcome question.

### 9.6. Relational Quantum Mechanics

Rovelli’s relational quantum mechanics (RQM) [13] offers one of the most radical and carefully articulated responses to the measurement problem. RQM holds that quantum states are not absolute properties of systems but are defined only relative to other physical systems. In this view, there is no observer-independent “state of the world”—all physical quantities are relational. When Alice measures particle A, the state of B changes *relative to Alice*, but the state of B *relative to Bob* (who has not yet interacted with either particle or with Alice) remains unchanged until Bob performs his own measurement or receives Alice’s result.

Our framework shares a key conclusion with RQM: nothing physical changes at B’s location when A is measured. Both approaches reject the idea of instantaneous nonlocal collapse as a physical process. Furthermore, both frameworks take seriously the idea that the description of a subsystem depends on the physical context—in RQM, this is the observer, in our framework, it is the factorizability of the global state.

However, the two approaches differ in their ontological commitments. RQM is ontologically deflationary: it denies that there is any absolute, observer-independent quantum state. Our ψ-ontic holism takes the opposite stance—the global wavefunction is objectively real and observer-independent. Where RQM dissolves the measurement problem by relativizing reality, we dissolve the nonlocality problem by globalizing it: reality is the holistic wavefunction, and the appearance of subsystem properties is emergent through factorization.

A further difference concerns the status of correlations. In RQM, the correlations between Alice’s and Bob’s results are established only when the two observers interact and compare results—there is no “fact of the matter” about the correlation prior to this comparison. In our framework, the correlations are objective features of the global wavefunction from the moment of preparation, encoded in its non-factorizable structure. What is deferred is not the reality of the correlations but our *access* to them: Alice and Bob can only verify the correlations by comparing results through a classical (local) channel.

Despite these differences, we regard RQM and ψ-ontic holism as complementary perspectives on the same underlying insight: that the apparent nonlocality of quantum mechanics reflects a failure to properly account for the relational or holistic structure of quantum states, rather than a genuine faster-than-light influence.

## 10. Discussion and Conclusions

### 10.1. Summary of the Argument

We have argued that the apparent nonlocality of quantum measurement arises from a category error: the false presupposition that subsystems of entangled systems possess independent ontic states. Our argument proceeds in four steps:

1. For entangled systems, the global wavefunction is the sole ontic object. Subsystem “states” (reduced density matrices) are calculational tools without independent ontological status.
2. Measurement is a local dynamical process. Concrete models (CSL, gravitational collapse, decoherence) show that collapse involves local modification of wavefunction components at the measurement site.
3. Local collapse causes factorization of the global state. Subsystem ontology emerges as a consequence of factorization, not as a pre-existing feature.
4. Bell’s theorem is respected, not evaded: factorizability (Bell local causality) fails, as it must. In our framework the failure is confined to outcome independence and is carried by the nonseparability of the global ontic state λ=|Ψ〉, while parameter independence—dynamical locality—holds exactly. Independently, the Kochen–Specker and GHZ theorems block the EPR inference from perfect correlations to noncontextual pre-existing values.

The conclusion is that quantum mechanics is nonseparable but not nonlocal. The fundamental ontology is holistic (the global wavefunction in configuration space), but the dynamics are local (measurement modifies only local components). Classical separability is emergent through decoherence.

### 10.2. The Central Distinction: Nonseparability vs. Nonlocality

Following Howard [8], we distinguish sharply between *nonseparability*—the denial that spatially separated systems always possess independent real states (a feature of the ontology)—and *nonlocality*—the claim that events at one location can instantaneously affect the physical state at a distant location (a feature of the dynamics).

Standard accounts conflate these two concepts. They observe that entangled systems are nonseparable (true) and conclude that measurement dynamics are nonlocal (not necessarily true). Our framework maintains nonseparability while denying nonlocality: the global wavefunction is holistic (nonseparable), but measurement dynamics are local (they modify only local components). Figure 3 situates our framework within the resulting taxonomy of interpretations.

### 10.3. Relation to the Companion Papers

This paper provides the ontological foundation for the program developed across three complementary works. The first [6] modeled decoherence-based measurement in the EPR scenario as an entirely local process; the corrected analysis in the second [5] shows that the outcome statistics in the measurement basis are preserved exactly, while the maximal CHSH value decays smoothly from the Tsirelson bound to the classical bound as decoherence completes—confirming that quantum correlations are structural features of the entangled state, locally consumed rather than nonlocally maintained. The second [5] further proposed Postulate M, establishing that a local measurement axiom is structurally consistent with quantum mechanics, no-signaling, and frame independence. The present paper explains *why* such local accounts succeed: because measurement is a local dynamical process acting on a holistic global wavefunction, and the appearance of nonlocal collapse is an artifact of falsely presupposing subsystem ontology.

Together, these three papers argue that quantum measurement can be fully understood as a local process within a holistic ontology, without invoking nonlocal influences, hidden variables, or modifications to the dynamics beyond those already motivated by the measurement problem.

### 10.4. The Status of Single-Particle Talk

A natural question (raised by a referee) is whether our framework renders the very concept of a single particle of a multi-particle entangled state meaningless. It does not. Subsystem A’s Hilbert-space factor, its local degrees of freedom, and its local observable algebra are perfectly well defined, and local dynamics couple to them—this is how Alice can measure A at all. What A lacks, while the global state is non-factorizable, is an independent *ontic state*: a self-standing “way A is” from which its measurement responses could be read off without reference to the whole. Single-particle talk therefore remains fully meaningful at the operational level ($\rho_{A}$ predicts every local statistic exactly) and becomes ontologically well-founded the moment the global state factorizes. The concept is not meaningless; it is *derivative*—emergent with factorization rather than fundamental.

### 10.5. Testable Consequences and Quantum-Simulation Prospects

Is the present proposal only a re-description of quantum mechanics, or does it have empirical content? We answer in two parts. Insofar as the framework re-reads standard quantum mechanics (with decoherence), its distinctive content is interpretive, and we state this plainly. However, the framework as developed across the companion papers is tied to the quantitative structure that is testable. First, the decoherence law $S_{\max}\left(t\right)=2\sqrt{1+{\left|z\left(t\right)\right|}^{2}}$ [5] is a falsifiable prediction for Bell tests performed on pairs subjected to controlled local dephasing before measurement: the maximal violation should track the independently measurable coherence |z(t)| along this specific curve, and the numerical realization of the full branch-selection dynamics is given in a companion study [43]. Second, if the single-outcome mechanism is CSL-like, the framework inherits the experimentally active program constraining collapse parameters. Third, the local branch-selection dynamics itself—effectively a nonunitary, norm-decreasing evolution of one component—is amenable to quantum simulation: duality-algorithm techniques for realizing nonunitary single-qubit operators on quantum hardware [44] provide a natural laboratory in which the factorization process of Section 4 could be simulated and its trajectory statistics checked against the martingale predictions of [5,43].

### 10.6. Open Questions

Several questions remain for future work:

(a) **Relativistic formulation.** Our use of CSL as illustration inherits CSL’s known difficulties with Lorentz covariance. A fully relativistic treatment of local collapse dynamics remains an open challenge, though promising approaches exist [45].
(b) **Gravitational connection.** If gravitational self-energy provides the mechanism for wavefunction collapse (Penrose–Diósi models), then our framework predicts a specific interplay between gravitational entanglement generation and gravitational factorization. This connection may yield experimentally testable predictions in the context of tabletop quantum gravity experiments [46,47].
(c) **The preferred basis problem.** Our account relies on environmental decoherence to select pointer states and drive factorization. The question of whether decoherence fully solves the preferred basis problem, or whether additional structure is needed, remains an active area of research.
(d) **Multipartite entanglement.** We have focused on bipartite entanglement. The extension to multipartite entangled states (GHZ, W, cluster states) and to continuous variable systems requires further development.

### 10.7. Conclusions

The quantum measurement problem has been complicated by the assumption that measurement on one part of an entangled system must nonlocally affect the other part. This assumption is a consequence of treating subsystem descriptions as ontologically fundamental. Once we recognize that for entangled systems, only the global wavefunction is real—subsystem ontology is emergent, not fundamental—the apparent nonlocality dissolves. Measurement is local. Correlations are structural, rooted in conservation laws—such as angular momentum conservation—that govern the preparation of entangled states. Separability is emergent. The quantum world is holistic but not, in any dynamical sense, nonlocal.

In this respect, our framework vindicates Einstein’s original vision in the EPR argument: that a complete physical theory should be both local and realistic. Einstein objected not to quantum mechanics’ predictions but to the apparent nonlocality implied by the standard interpretation. Our ψ-ontic holism preserves realism (the global wavefunction is real) and locality (measurement dynamics are local), achieving the reconciliation that Einstein sought, though in a form he might not have anticipated, since the “reality” in question is the holistic global wavefunction rather than the separable local states he envisioned.

## Data Availability

All analytical content relevant to this work is presented within the paper. No additional data were generated.
